# Korean adolescents’ coping strategies on self-harm, ADHD, insomnia during COVID-19: text mining of social media big data

**DOI:** 10.3389/fpsyt.2023.1192123

**Published:** 2023-11-15

**Authors:** Ryemi Do, Soyeon Kim, You Bin Lim, Su-Jin Kim, Hyerim Kwon, Jong-Min Kim, Sooyeon Lee, Bung-Nyun Kim

**Affiliations:** ^1^Biomedical Research Institute, Seoul National University Hospital, Seoul, Republic of Korea; ^2^Department of Psychiatry and Behavioural Neurosciences, McMaster University, Hamilton, ON, Canada; ^3^Waypoint Research Institute, Waypoint Centre for Mental Health Care, Penetanguishene, ON, Canada; ^4^Division of Child and Adolescent Psychiatry, Department of Neuropsychiatry, Seoul National University Hospital, Seoul, Republic of Korea; ^5^VAIV Company, Seoul, Republic of Korea

**Keywords:** adolescent, text mining, social media, self-harm, insomnia, attention, COVID-19, South Korea

## Abstract

**Introduction:**

Since the Coronavirus disease 2019 (COVID-19), public safety measures, including social distancing and school closures, have been implemented, precipitating psychological difficulties and heightened online activities for adolescents. However, studies examining the impact of the pandemic on adolescent mental health and their coping strategies in Asian countries are limited. Further, most studies have used survey measures to capture mental health challenges so far. Accordingly, this study aimed to examine the psychological challenges South Korean adolescents experienced and their coping strategies during the pandemic using the Natural Language Processing (NLP) and Text mining (TM) technique on adolescents’ social media texts/posts.

**Methods:**

The data were gathered from social media texts/posts such as online communities, Twitter, and personal blogs from January 1, 2019, to October 31, 2021. The 12,520,250 texts containing keywords related to adolescents’ common psychological difficulties reported during the pandemic, including self-harm, Attention-Deficit/Hyperactivity Disorders (ADHD), and insomnia, were analyzed by TM, NLP using information extraction, co-occurrence and sentiment analysis. The monthly frequency of the keywords and their associated words was also analyzed to understand the time trend.

**Results:**

Adolescents used the word “self-harm” in their social media texts more frequently during the second wave of COVID-19 (August to September 2020). “Friends” was the most associated word with “self-harm.” While the frequency of texts with “Insomnia” stayed constant throughout the pandemic, the word “ADHD” was increasingly mentioned in social media. ADHD and insomnia were most frequently associated with ADHD medications and sleeping pills, respectively. Friends were generally associated with positive words, while parents were associated with negative words.

**Conclusion:**

During COVID-19, Korean adolescents often expressed their psychological challenges on social media platforms. However, their coping strategies seemed less efficient to help with their difficulties, warranting strategies to support them in the prolonged pandemic era. For example, Korean adolescents shared psychological challenges such as self-harm with friends rather than their parents. They considered using medicine (e.g., sleeping pills and ADHD medication) as coping strategies for sleep and attention problems.

## Introduction

In the face of a prolonged Coronavirus disease 2019 (COVID-19) timeline, adolescents often experience loneliness, depression, and anxiety (1–3). In addition to emotional problems, adolescents experience more attention (4) and sleep problems (5). However, there is little research on South Korean adolescents and what coping strategies adolescents pursued during the pandemic. Further, most studies used self-reported measures to assess mental health challenges, which entail inherent bias (4, 6). Analyzing social media data using text mining and natural language processing methods can provide a more objective and naturalistic understanding of how Korean adolescents perceive and cope with mental health challenges during the pandemic.

The COVID-19 dramatically changed the lives of adolescents. In most countries, online classes were held due to school closures, and adolescents’ daily routines became irregular. During the pandemic, adolescents frequently reported difficulty concentrating and sleeping (4, 5), and suicide-related behavior increased (7). However, recent findings on suicide-related behavior and sleep during the pandemic are inconsistent (8). For example, while suicide-related behaviors increased among adolescents in Sweden, suicidal thoughts and attempts decreased among Korean adolescents during the pandemic (9). Similarly, while some studies reported increased sleep time (10, 11) and enhanced sleep quality, such as better sleep satisfaction (12, 13), some indicated no change in sleep time (12) and even exacerbated sleep quality, such as insomnia, and sleep disturbance (14, 15). Another common challenge experienced among adolescents during the pandemic is attention problem. Many studies reported that attention problems intensified during COVID-19, and in particular, adolescents with Attention-Deficit/ Hyperactivity Disorder (ADHD) had difficulty adapting to the changed educational environment and life during COVID-19 (16, 17). However, most studies were conducted at a specific point or during a short period, overlooking the general time trend. For example, a study in Sweden suggested that self-harm increased during COVID-19 (18). However, whether it increased immediately after or later during the pandemic remains unclear (18). Therefore, investigating a longer-term trend in adolescents’ perception of mental health needs and coping strategies is needed. In addition, understanding how adolescents cope with mental health challenges is crucial to strategize in the ways to support them (19). However, there is dearth of studies investigating the source of help-seeking for adolescents.

In Korea, most adolescents are vastly engaged in social media activities (6). Even before the pandemic, Korean adolescents engaged in many internet-related leisure activities. For example, 80% of adolescents use social media (20), and used smartphones for about three and a half hours daily, mainly for messages or SNS (21). Analyzing adolescents’ discourse on social media reflects how adolescents perceived and coped with their mental health difficulties during the pandemic. South Korean government took stringent, centralized policy directions such as the enforcement of working at home, prohibition of face-to-face gatherings, and public places closings (22, 23). All elementary, middle and high schools in South Korea were temporarily closed from March 2020 to June 2020, then switched to online classes. All the schools opened gradually, combining online classes in 2020 (24, 25). Specifically, stepwise school opening was implemented, in which face-to-face and online classes were alternately held for each grade level. For example, students were only allowed to go to school for a few days a week, and talking in the classroom was prohibited even while wearing a mask. Therefore, face-to-face contact among Korean adolescents has been limited, and their social media activities have increased exponentially (6).

Analyzing the trend of adolescents’ mental health-related discourse on social media may be an optimal way to understand how adolescents perceived their mental health and coped with their mental health needs during the pandemic. Using text-mining (TM) analysis on social media texts that reflect adolescents’ genuine experiences can address the social-desirability bias inherent to survey-based studies (26, 27). TM is the process of extracting digitized information from language texts into numbers that computers can understand through natural language processing (NLP). TM method enables investigating whether specific keywords or texts increase or decrease over time, what emotions appear most often over time (sentiment analysis), and what related words the keywords appear with (co-occurrence analysis). Therefore, text mining analysis on social media data analysis can be an alternative approach to the conventional self-report or qualitative approach. For example, a British study showed that young people (15–24 years) experienced depression and anxiety during the epidemic using the qualitative method of ethnographic techniques on social media (28). Qualitative research methodologies like thematic analysis are relatively less objective than text mining as this approach takes researchers’ collective and subjective viewpoint in the analysis (29). TM approach can be more objective in identifying a trend of mental health needs and coping strategies by extracting and decomposing information from large volumes of social media posts during the pandemic (30). Recently, a few studies applied text mining techniques to social media big data to examine mental health challenges during the pandemic (31, 32). Valdez et al. used TM techniques to investigate the emotional valence of US tweets during the pandemic, (i.e., stay-at-home mandates) and found a trend of negative sentiment (31). Zhang et al. (32) showed that adolescents with mental health concerns were more sensitive to the pandemic. They posted more comments on Reddit communities just after the significant pandemic events (32). In a recent study, Seo et al. analyzed barriers to psychiatric help-seeking in South Korea with social media data (33). Authors found that co-keywords of “barriers” were structural discrimination, public pre-service, adverse drug effects, and low accessibility. However, most of the previous studies that used text mining analysis on mental health-related topics during the COVID-19 era only provided information on how positive/negative emotions increased or decreased over time in the general sample, and studies focusing on adolescents are limited. Further, previous studies were mainly conducted in English-speaking countries such as the United States (28).

To address the research gap, the current study used TM and NLP -based co-occurrence analysis on social media texts posted by South Korean adolescents during the pandemic The present study sought to explore how Korean adolescents experienced and coped with most prevalent mental health problems, such as self-harm, ADHD, and insomnia based on their discourse on social media during the pandemic. Additionally, we examined the monthly trends of social media posts/texts frequency on commonly reported mental health challenges during the pandemic keywords such self-harm, ADHD, and insomnia.

## Materials and methods

### Data source and collection

Social media data for the current research were collected and processed by a social media analytics company in South Korea (VAIV).^3^ Details of the data management and analysis can be found in Seo et al. (33). Texts (posts or comments) shared between January 1, 2019, and October 31, 2021, on social network services (SNS) such as Twitter, online communities, personal blogs were collected. In Korea, online communities, such as internet forums and ‘Cafes’ - Naver^2^ or Daum^3^ - and online platforms^4^,^5^ are actively used among adolescents. Anyone can create and operate a “Cafe” in portal sites such as Naver and Daum, and tens of thousands of people with the same interests join the cafe online. We also included Twitter, which is widely used among Korean adolescents.

### Data extraction

To extract the relevant text from social media, the research team listed filter-in-terms related to adolescent mental health used in the previous study on mental health (33). The 145 filter-in-terms (Supplementary File) are composed of keywords related to adolescent’s mental health, such as ‘teacher,’ ‘exam,’ ‘class,’ ‘study,’ ‘mood,’ ‘depression,’ ‘ADHD,’ ‘insomnia,’ ‘school counselling,’ and ‘Weeclass’ (a school counselling program in South Korea). As a result, we obtained a total of 43,123,953 posts, including 19,066,634 tweets (44.2%), 4,643,281 personal blog posts (10.8%), and 19,414,038 online community site posts (45.0%), and a total of 12,520,250 adolescents-related texts were included. In order to confirm that adolescents wrote posts, we checked whether adolescent-related keywords appeared on the posts. Suppose users of online communities shared keywords which only adolescents use in a Korean context, such as information on a university entrance exam, midterm exam or school violence, the online communities were deemed adolescents’ communities. Research team visited each online community checking the posts to confirm if the online community belong to adolescents.

### Data processing

**Figure 1:**
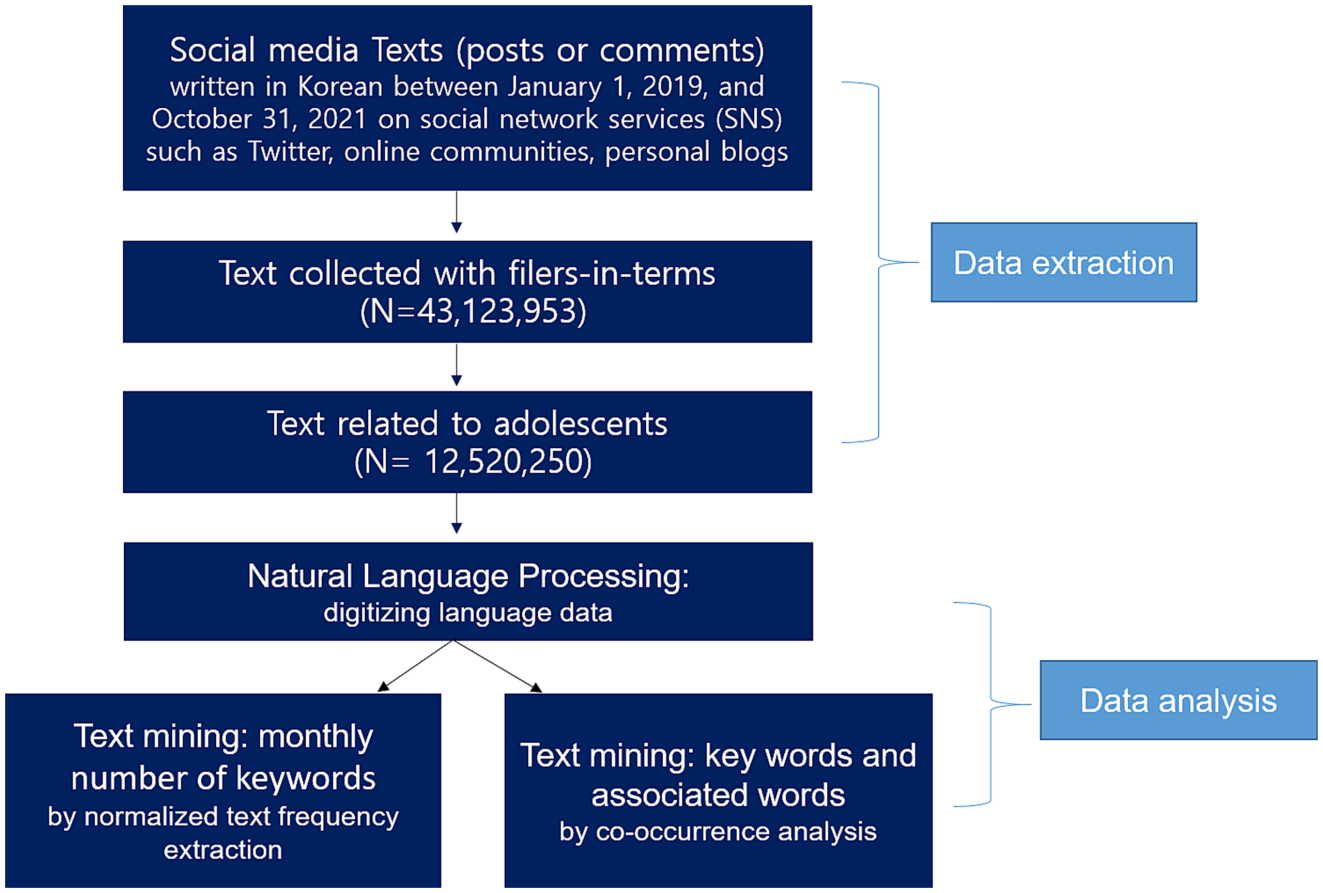
Flow chart of framework and texts in this study.

The extracted data was analyzed using NLP and TM techniques (Figure 1). NLP is the process of digitizing actual natural language texts - digitizing all morphemes in a text or grammatically classifying all words in the document. Texts were digitized into the minimum unit of words, that is, morphemes. Any grammar that classifies all words in a text is also converted into numbers using language dictionaries of words and grammar.

TM is a process of extracting information from the text in detail. Words were classified by ‘keyword category classification’ (Table 1). The top conceptual categories included health response, mental health, relation and predicate. ‘A predicate word’ is an adjective or verb in a sentence showing what the subject is doing or what the subject is. The number of keywords such as self-harm, ADHD, and insomnia appearing in the social media text by month was calculated by the ‘Normalized text Frequency Extraction’ technique and presented as a graph. We avoided overestimating word frequency by normalizing the frequency of texts instead of the term frequency. This method prevents the calculation of the number of texts, including keywords, from being biased because a specific word appears too much in one person or a specific text. In order to know how many texts with the keyword there are in total, it was counted as one even if the word appeared several times in one text. For example, even if a word such as ‘self-harm’ appears multiple times within a text, the text’s frequency is counted as one. In this study, we calculated ‘text frequency,’ the number of texts containing the words self-harm, ADHD, and insomnia monthly, and presented a frequency trend graph of when the number of texts changed. Normalized text frequencies of the individual and associated keywords were extracted from the source texts. We could investigate the voices of adolescents using the normalized text frequencies per month for 2 years and 10 months, combined with the keyword taxonomy.

**Table 1:** Keywords categories.

Major category	Subcategory	Keywords
Health response	Agency	Medicine, psychiatry …
	Self-response	Diet, mindfulness …
	Etc	Vitamin …
Mental health	Mental illness diagnosis	ADHD, depressive disorder …
	Symptom	Appetite, fatigue, dizziness …
Relation	Age	12 years old, elementary school, middle school …
	Object	Mom, parent, friend, sister …
Predicate words (adjective & verb)	Positive	Good, fine, helpful …
	Negative	Dislike, difficult, bad …
	Neutral	Big, none, young …
	Verb	See, tough, go …

Another text mining method is the process of extracting information about relationships between words from texts based on the frequency of co-occurrence keywords. Co-occurrence analysis quantifies the degree of association between words in a text. It is a method of counting the association frequency as 1 when one keyword ‘appears together’ with the other keyword in the same text. Suppose in a text, ‘ADHD medications’ is mentioned more often than ‘counseling’ as an adjoining word for ADHD. In that case, the associated keyword, co-keyword for ADHD, is ‘ADHD medications,’ suggesting that attention problems are being dealt with by taking ADHD medications. The co-occurrence of two noun keywords is counted as one association when they appear together throughout the text. However, in the case of a predicate, the co-occurrence is counted only when a predicate appears in one sentence with a noun keyword. By checking which predicates and nouns are frequently mentioned together in a sentence within texts, we can figure out in which contexts the nouns are often mentioned. For example, when analyzing which predicate the word self-harm usually appeared with, it was found that the predicate “caught” often appeared together with the word self-harm and the predicate “caught” was often mentioned along with the word “parent.” In this case, it can be assumed that parents caught adolescents’ self-harm. Sentiment analysis analyzes texts through the frequency of occurrence of keywords classified as positive and negative. In a text, we examined whether adjectives with parents are positive or negative and whether adjectives with friends are positive or negative. Sentiment analysis is one of the co-occurrence analysis methods that examines a predicate word (an adjective or verb) based on sentiment. It classifies whether a predicate is a positive or negative sentiment and examines which positive or negative predicates are associated with keywords. Estimating how adolescents perceive parents or friends is possible by looking at which positive/negative adjectives or verbs are related, respectively.

## Results

### Self-harm

**Figure 2:**
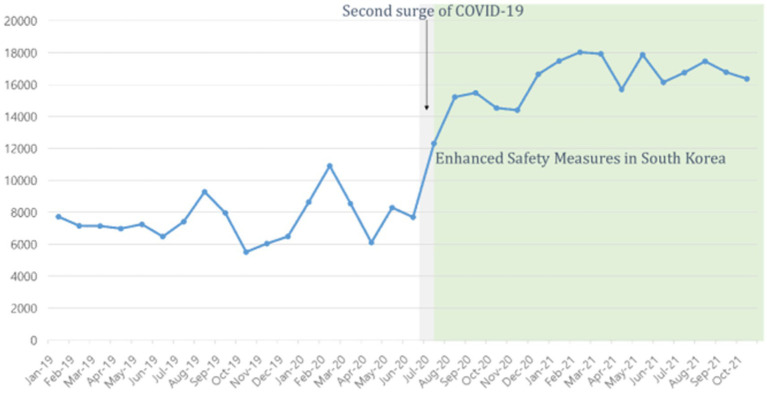
Monthly frequency of self-harm-mentioned texts on Twitter.

The word ‘self-harm’ (‘Jahae’ in Korean) was frequently mentioned on Twitter, blogs, and online communities in South Korea. Self-harm was mostly mentioned on Twitter than in other online communities. Texts containing the word ‘self-harm’ were extracted monthly according to the normalized text frequency extraction method. References to self-harm on Twitter increased from 2019 to 2021 and spiked sharply in the summer of 2020 (Figure 2) during the second surge of COVID-19 when the Korean government strengthened safety measures. Text including word of self-harm did not increase in the beginning of COVID-19, increased since the August of 2020. ‘People,’ and ‘friends’ were the most frequently associated words with self-harm, and their frequency increased during the pandemic. On Twitter, ‘see’ and ‘upload’ were ranked high in predicate words (verb or adjective) related to ‘self-harm & people’ (Table 2). ‘See’ and ‘know’ were ranked high in predicate words (verb or adjective) related to ‘self-harm & friend’ (Table 2). Between 2019 and 2021, the number of mentions of ‘self-harm & being caught’ on Twitter increased. Therefore, we ranked the co-keywords of ‘self-harm & get caught’ in the ‘relationship’ category on Twitter as co-occurrence analysis. Among the words related to ‘self-harm & caught’ in the ‘relationship’ category on Twitter, ‘mom,’ ‘parent,’ and ‘friend’ were the highest in order (Table 2). Based on this, we can assume that self-harm was shared online and with friends, but not with the parents (there were many expressions such as ‘parents caught self-harm’, meaning ‘the parents discovered self-harm’).

**Table 2:** Associated words ranking of ‘self-harm’ in Twitter of South Korea (in 2019–2021).

	Word (*in Korean*) | text frequency					
Ranking	Predicate words associated with ‘self-harm and people’		Predicate words associated with ‘self-harm and friend’		Words in ‘relation’ category associated with ‘self-harm and get caught’	
1	See (*boda*)	523	See (*boda*)	526	Mom (*eomma*)	640
2	Upload (*ollida*)	415	Know (*alda*)	432	Parent (*bumonim*)	256
3	Know (*alda*)	320	Do not exist (*eobsda*)	327	Friends (*chingu*)	224
4	Hard (*himdeulda*)	285	Go (*gada*)	258	Father (*appa*)	151
5	Think (*saeng-gaghada*)	225	Hard (*himdeulda*)	226	Person (*salam*)	141
6	Do not exist (*eobsda*)	219	Do not know (*moleuda*)	212	Teacher (*seonsaengnim*)	94
7	Do not know (*moleuda*)	208	Meet (*mannada*)	206	Child (*ai*)	80
8	Live (*salda*)	160	Upload (*ollida*)	186	Family (*gajog*)	77
9	Die (*jugda*)	147	Show (*boida*)	166	Older sister (*eonni*)	51
10	Be seen (*boida*)	141	Come (*oda*)	164	Brother (*dongsaeng*)	45

### ADHD

**Figure 3:**
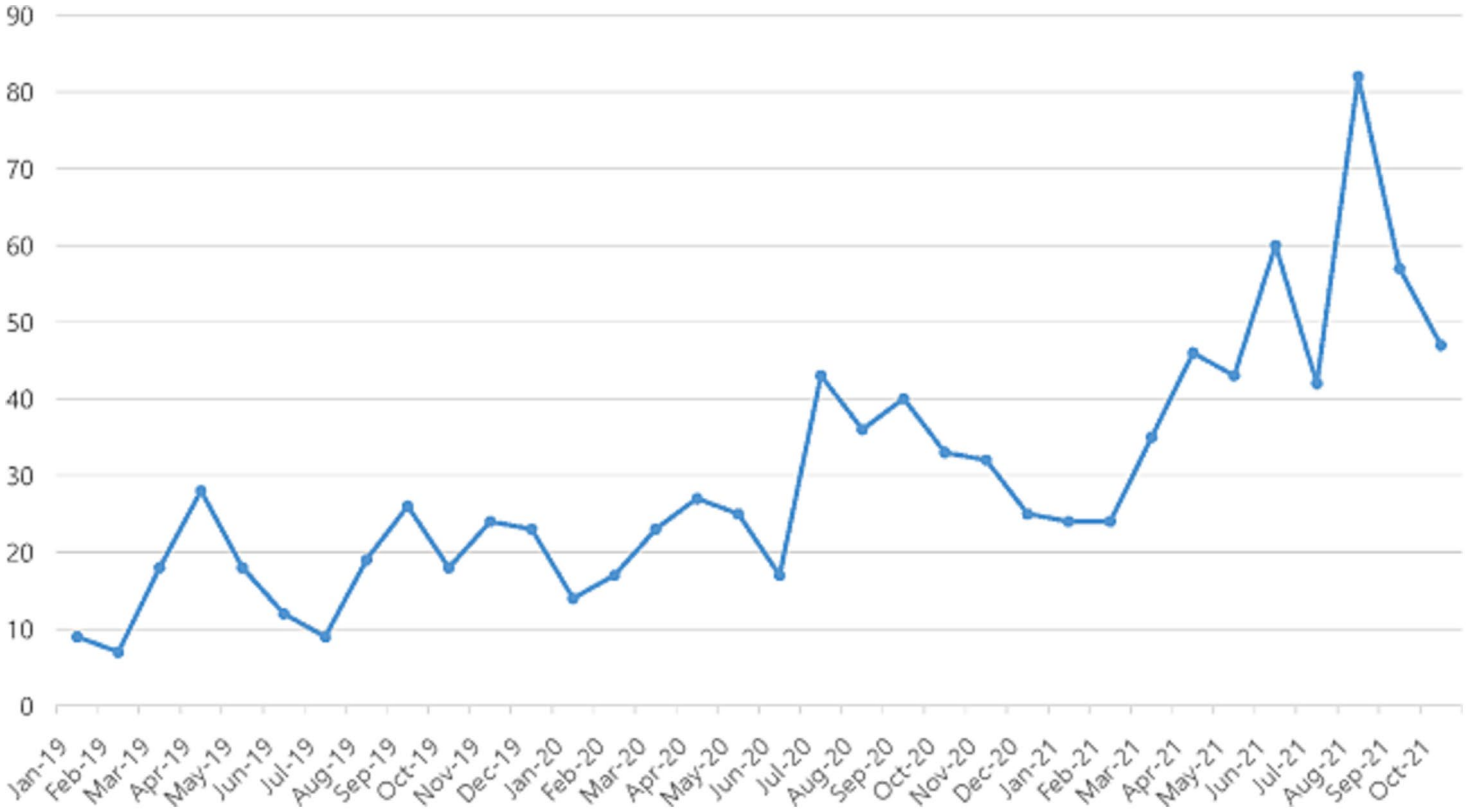
Monthly frequency of ADHD-mentioned texts on adolescents’ online communities.

From January 2019 to October 2021, texts containing words such as attention disorder, attentional disorder, and attention deficit disorder or ADHD, which refer to ADHD, were extracted monthly using the normalized text frequency extraction method. Mentions of ADHD continued to increase from the start of COVID-19 until October 2021 (Figure 3). When ranked the co-keywords of ADHD by year as co-occurrence analysis, we found that ‘ADHD medications’ was most frequently mentioned as an associated word for ADHD. Among the associated words of ADHD, the ranking of ADHD medications heightened during COVID-19 (Table 3). ‘ADHD medications’ was ranked 10th in 2019, 7th in 2020 and 1st in 2021.

**Table 3:** Associated words ranking of ‘ADHD’ in the adolescents’ communities.

Top ten words most frequently associated with ‘ADHD’ in the adolescents’ online communities in South Korea (in 2019–2021).						
Word (*in Korean*) | text frequency						
Ranking	2019		2020		2021	
1	Study (*gongbu*)	59	Study (*gongbu*)	75	Medication (*yag*)	86
2	Thought (*saeng-gag*)	50	Time (*sigan*)	55	Study (*gongbu*)	80
3	Person (*salam*)	46	Person (*salam*)	55	Person (*salam*)	71
4	Now (*jigeum*)	42	Now (*jigeum*)	51	Receive (*badda*)	68
5	Words (*mal*)	41	Depression (*uuljeung*)	49	Concentration (*jibjung*)	65
6	Time (*sigan*)	38	The ability to concentrate (*jibjunglyeog*)	46	Time (*sigan*)	59
7	Concentration (*jibjung*)	33	Medication (*yag*)	46	The ability to concentrate (*jibjunglyeog*)	56
8	School (*haggyo*)	33	Concentration (*jibjung*)	44	Words (*mal*)	56
9	Child (*ai*)	33	Receive (*badda*)	44	Now (*jigeum*)	51
10	Medication (*yag*)	33	Problem (*munje*)	43	Go (*gada*)	50

### Sleep problem: insomnia

**Figure 4:**
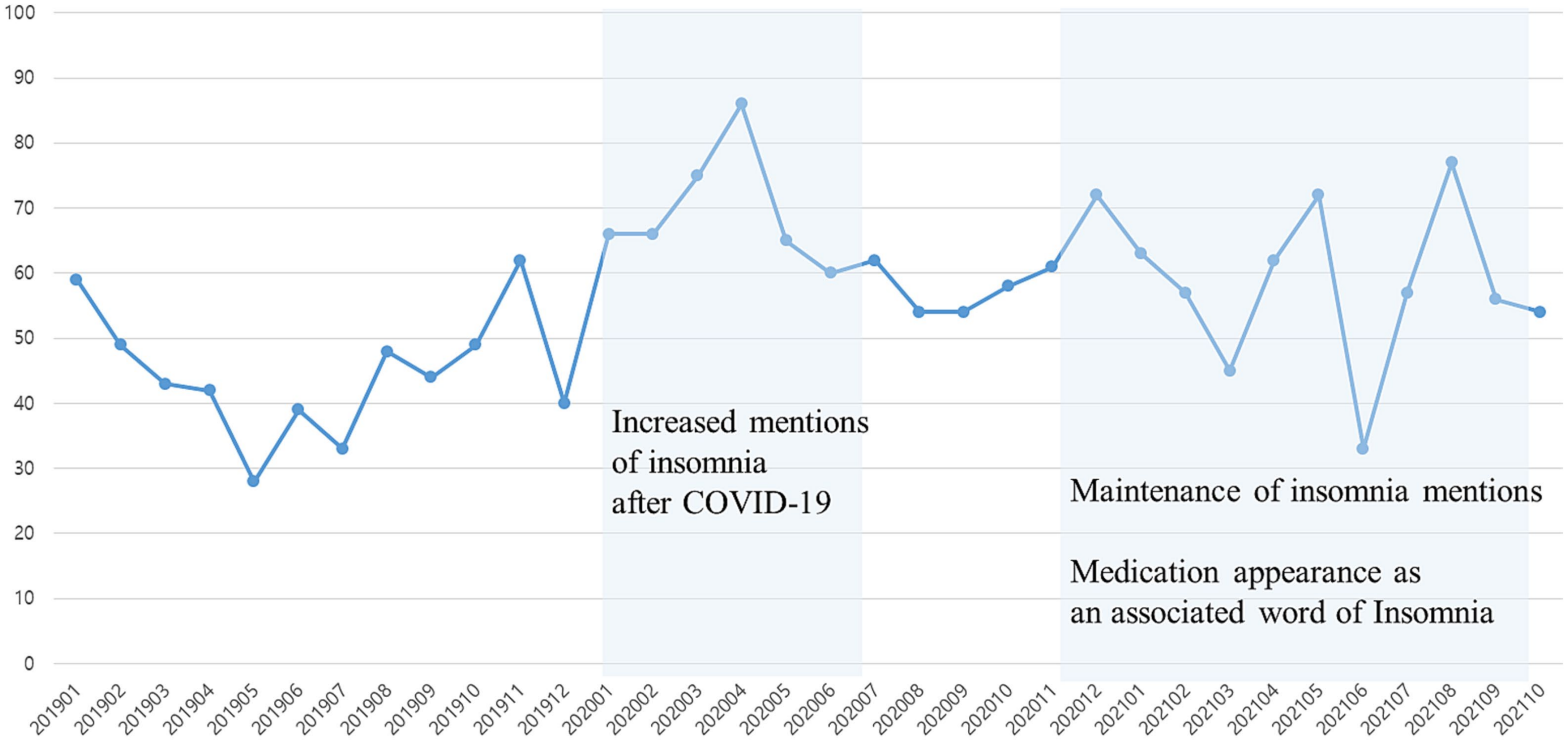
Monthly frequency of insomnia-mentioned texts on adolescents’ online communities.

In April 2020, when COVID-19 started to spread, the mention of ‘insomnia’ surged (Figure 4). When we ranked the co-keywords of insomnia in the category of health response as co-occurrence analysis, ‘Medication’ and ‘sleeping pills’ were the first and second frequently mentioned associated words of insomnia (Table 4). During COVID-19, the mention of insomnia has increased, along with the associated word ‘medication.’

**Table 4:** Associated words ranking of ‘Insomnia’ in the category of ‘Health Response’.

Top ten words that were most frequently associated with ‘Insomnia’ in the category of ‘Health Response’ category in the adolescents’ online communities in South Korea (in 2019–2021)		
Ranking	Word (*in Korean*) | text frequency	
1	Medication (*yag*)	154
2	Sleeping pills (*sumyeonje*)	97
3	Psychiatry (*jeongsingwa*)	64
4	Advice (*jo-eon*)	50
5	Cure (*chilyo*)	47
6	Exercise (*undong*)	41
7	Side effect (*bujag-yong*)	32
8	Prescription (*cheobang*)	24
9	Cause (*won-in*)	23
10	Consolation (*wilo*)	22

### Help-seeking source of adolescents’ mental health problem

As associated words of adolescents’ mental health response category for mental disease, the words “medicine,” “psychiatry,” and “treatment” are most frequently mentioned. When looking at the degree to which mental health-related words appear with teachers in the text, 65% of texts mentioned mental health-related words with homeroom teachers and 25% with counselling teachers. Adolescents mentioned homeroom teachers rather than counselling teachers when talking online about mental health.

We analyzed the ranking of predicate words (adjective or verb) that frequently appear together with parents or friends and identified whether associated predicate words were positive or negative according to the sentiment analysis method. Based on discourse in adolescents’ online communities, there were more positive words related to friends than negative words, while there were more negative words related to parents than positive words (Table 5). In the case of associated words of a friend, positive words accounted for 45.2%, negative words accounted for 54.8%, and the top two ranked associated words were ‘liking’ and ‘good.’ In the case of parent-related words, the percentage of positive words was 30.6%, and those of negative words were 69.4%, and ‘difficult’ and ‘no’ were ranked first and second. Furthermore, mentions related to friends were more than three times more frequent than mentions related to parents. Adolescents perceived friends more positively than parents.

**Table 5:** Associated words ranking with friends and parents in the category of positive/negative emotions.

Word (*in Korean*) | text frequency							
Ranking	Words associated with friends	Positive/negative	Frequency	Ranking	Words associated with parents	Positive/negative	Frequency
1	Like (*joh-ahada*)	Positive	19,822	1	Difficult (*himdeulda*)	Negative	5,604
2	Good (*johda*)	Positive	15,389	2	Do not become (*andoeda*)	Negative	3,909
3	Difficult (*himdeulda*)	Negative	14,591	3	Good (*johda*)	Positive	3,686
4	Close (*chinhada*)	Positive	12,902	4	Fight (*ssauda*)	Negative	3,417
5	Be good at (*jalhada*)	Positive	8,924	5	Cannot (*moshada*)	Negative	2,964
6	Dislike (*sihda*)	Negative	8,643	6	Dislike (*silhda*)	Negative	2,680
7	Beautiful (*yeppeuda*)	Positive	8,403	7	Sorry (*joesonghada*)	Negative	2,564
8	Do not become (*andoeda*)	Negative	7,925	8	Be good at (*jalhada*)	Positive	2,431
9	Cannot (*moshada*)	Negative	7,571	9	Help (*dobda*)	Positive	2,371
10	Be seen (*boida*)	Positive	7,531	10	Be seen (*boida*)	Positive	2052
11	Help (*dobda*)	Positive	6,910	11	Like (*joh-ahada*)	Positive	2007
12	Fight (*ssauda*)	Negative	6,831	12	Want to go (*gagosipda*)	Positive	1986
13	Do not’ like (*sih-eohada*)	Negative	5,690	13	Not good (*johjianhda*)	Negative	1974
14	Fine (*gwaenchanhda*)	Positive	5,408	14	Oppose (*bandaehada*)	Negative	1959
15	Irritated (*jjajeungnada*)	Negative	5,100	15	Scary (*museobda*)	Negative	1,537
16	Not good (*johjianhda*)	Negative	4,708	16	Be caught (*geollida*)	Negative	1,531
17	Suffer (*danghada*)	Negative	4,512	17	Fine (*gwaenchanhda*)	Positive	1,513
18	Laugh (*usda*)	Positive	4,434	18	Suffer (*danghada*)	Negative	1,343
19	Funny (*usgida*)	Positive	4,241	19	Want to die (*juggosipda*)	Negative	1,244
20	Sorry (*mianhada*)	Negative	3,945	20	Irritated (*jjajeungnada*)	Negative	1,203

## Discussion

The current study explored adolescents’ trends of mental health needs and coping strategies using social media big data analysis during COVID-19. Our findings suggest that self-harming thoughts and behaviors were prevalent during the pandemic among adolescents, showing an increasing trend during the second wave of COVID-19 (August to September 2020). Korean adolescents coped with self-harming thoughts/behaviors by sharing them online or by talking to their peers. Further, although ADHD-related texts continued to increase since the beginning of COVID-19, insomnia-related texts remained constant. ‘Sleeping pills’ and ‘ADHD medication’ were the most associated words of sleep and attentional problems in adolescents. Korean adolescents may consider medications as coping strategies when they experience sleep and attention problems. Associated words of mental health problems were homeroom teachers rather than counselling teachers, even though counselling teachers specialized in mental health in Korean school.

Our findings indicate that self-harm thoughts and behaviors were shared online among friends but hidden from parents. Adolescents often share self-harm thoughts and behavior online as a coping strategy. Parents were the associated word for ‘self-harm and being caught.’ Therefore, it may be speculated that adolescents want to hide their self-harm thoughts and behaviors from their parents. Our findings align with previous studies suggesting self-harming behavior is primarily disclosed to friends (34). Based on our findings, suicide prevention interventions that leverage peer relationships, such as the “Sources of Strength Program” (35), may be helpful. Further, parent education programs that fortify communication skills with adolescents are recommended. When psychiatrists provide treatment, it is necessary to inform adolescents’ parents and provide guidance and education on how to respond, as adolescents may hide suicidal thoughts or behaviors from their parents. We also found that discourse on self-harm doubled on Twitter in the summer of 2020, at the second surge of COVID-19 among Korean adolescents. Increased mentions of self-harm continued until October 2021. Our finding is interestingly in line with a previous study reporting that Japan’s monthly suicide rate increased by 16% during the second wave (July–October 2020), while the monthly suicide rate decreased by 14% during the first 5 months of the pandemic (February–June 2020) (36). However, some Korean studies reported a decrease in suicide-related behavior in 2020 (9, 37). Considering the results of this study and the existing research results showing that self-harm worsens in the mid to late stages of a disaster (36, 38), preventative intervention efforts for community adolescents on suicide-related behavior are critically warranted.

This study showed that adolescents considered medication such as sleeping pills or ADHD medications as coping strategies for attention and sleep problems. Perhaps irregular lifestyles caused by school closures and increased online classes have in part, caused sleep and attention problems during the pandemic. Numerous studies have demonstrated lifestyle changes since the pandemic, including decreased physical activity and increased sedentary behavior, which could influence their sleep and psychological conditions (39, 40). Relatedly, our findings indicate that the comment of insomnia surged during the initial phase of COVID-19. Also, it is concerning that adolescents seem more likely to cope with attention problems, mainly by taking ADHD medications. It may be worth noting that these results do not necessarily mean that many Korean adolescents actually take sleeping pills or ADHD medications offline. In South Korea, adolescents cannot easily obtain sleeping pills or ADHD medications without a doctor’s prescription, and the substance abuse prevalence among Korean adolescents is relatively low (41). Rather, our results may indicate that adolescents consider sleeping pills and ADHD medications to cope with attention or sleep problems, suggesting a potential increase in substance-related problems in South Korea. Consuming drugs or medication to feel better is defined as risky coping, and risky coping plays a negative role in mental health while problem-analyzing and support-seeking coping play a positive role (42). Taken together, education about the pros and cons of medication is needed, including constructive ways to improve attention and build long-term healthy sleep habits such as sleep hygiene, the importance of stable daily routines, learning relaxation and stress management skills (43, 44), including problem-analyzing and support-seeking coping. Considering adolescents’ familiarity with online content, providing online education programs to teach mental health literacy and constructive coping strategies would be helpful.

Lastly, homeroom teachers were mentioned more commonly than school counselor in relation to mental health problems, which may imply that homeroom teachers are more approachable to adolescents with psychological difficulties than counseling teachers. Although this phenomenon may be limited to the Korean context, providing further training for homeroom teachers about adolescent mental health might be important to support and aid students when they seek help for mental health difficulties. Considering the results of this study, which suggests that adolescents are more likely to ask for help from homeroom teachers or friends who are closer to them, it may be necessary to focus mental health-related interventions on homeroom teachers, peer group campaigns, or peer counseling.

### Limitations

The current study has several limitations. First, we analyzed the data from social media platforms where people post their comments anonymously. Therefore, we could not derive exact demographic information of those who posted the texts extracted in this study (e.g., age, sex). Although we ensured that we extracted words used by adolescents and researchers checked the texts and online communities, due to privacy concerns, we could not verify the precise demographic information of those who posted the texts.

Further, our study’s findings cannot reflect all adolescents’ opinions, as some adolescents may be reluctant to engage in online communities. In future studies, it is necessary to study the characteristics and tendencies of active online adolescents. In addition, even among adolescents who are active in social media, there can be a difference between the actual experiences of adolescents and their experiences described in social media. Adolescents may not post their genuine feelings or perceptions on social media, especially in online communities where they can be open to others or friends. Further research is needed on which adolescents are active online and how adolescents differ in their online discourse and offline experiences.

The monthly frequency of texts, including self-harm, ADHD, and insomnia was presented in a graph. This study only showed the time of increased mentions, whether it increased immediately after the pandemic outbreak or in the middle of the pandemic. However, we did not statistically assess the significance of the increase in keywords during COVID-19 and the relation between monthly text frequency trends and COVID-19-related events.

## Conclusion

The current study used big data analysis on social media texts to explore how adolescents experienced mental health struggles and coped with them during the pandemic. This study suggests that adolescents experience mental health challenges in the prolonged pandemic era, and are more likely to ask for help from their homeroom teacher or friends who are closer to them, and they may be more likely to seek out medications to cope with mental challenges. It is essential to develop psycho-education and guidance for mental health problems for adolescents, such as self-harm, sleep and attention problems. The use of online programs, considering their massive use of SNS, may be helpful.

## Data availability statement

The raw data supporting the conclusions of this article will be made available by the authors, without undue reservation.

## Author contributions

B-NK conceptualized and supervised the study after funding acquisition. RD conceived the study and drafted the manuscript. YBL and SL revised the manuscript. J-MK collected the data and performed the data analyses. S-JK and HK provided administrative support and assisted in the study’s design. SK critically reviewed and edited the manuscript. All authors contributed to data analysis, drafting and revising the article, gave final approval of the version to be published, agreed on the journal to which the article has been submitted, and agreed to be accountable for all aspects of the work.

## Footnotes

^1^http://vaiv.kr

^2^http://www.naver.com

^3^http://www.daum.net

^4^https://www.dcinside.com/

^5^https://pann.nate.com/
